# Resting States Are Resting Traits – An fMRI Study of Sex Differences and Menstrual Cycle Effects in Resting State Cognitive Control Networks

**DOI:** 10.1371/journal.pone.0103492

**Published:** 2014-07-24

**Authors:** Helene Hjelmervik, Markus Hausmann, Berge Osnes, René Westerhausen, Karsten Specht

**Affiliations:** 1 Department of Biological and Medical Psychology, University of Bergen, Bergen, Norway; 2 Department of Psychology, Durham University, Durham, United Kingdom; 3 Bjørgvin District Psychiatric Centre, Haukeland University Hospital, Bergen, Norway; 4 Division of Psychiatry, Haukeland University Hospital, Bergen, Norway; 5 Department of Medical Engineering, Haukeland University Hospital, Bergen, Norway; Universiteit Gent, Belgium

## Abstract

To what degree resting state fMRI is stable or susceptible to internal mind states of the individual is currently an issue of debate. To address this issue, the present study focuses on sex differences and investigates whether resting state fMRI is stable in men and women or changes within relative short-term periods (i.e., across the menstrual cycle). Due to the fact that we recently reported menstrual cycle effects on cognitive control based on data collected during the same sessions, the current study is particularly interested in fronto-parietal resting state networks. Resting state fMRI was measured in sixteen women during three different cycle phases (menstrual, follicular, and luteal). Fifteen men underwent three sessions in corresponding time intervals. We used independent component analysis to identify four fronto-parietal networks. The results showed sex differences in two of these networks with women exhibiting higher functional connectivity in general, including the prefrontal cortex. Menstrual cycle effects on resting states were non-existent. It is concluded that sex differences in resting state fMRI might reflect sexual dimorphisms in the brain rather than transitory activating effects of sex hormones on the functional connectivity in the resting brain.

## Introduction

Sex differences in the brain have been shown on the structural, functional, and behavioral level, and partly reflect the sex-hormonal organization of the brain during early ontogenesis [for reviews on sexual dimorphisms see 1], [2]. Several studies suggest sex differences in functional brain organization. For example, it is widely assumed that women are generally more bilaterally organized than men [3]–[5], [but see 6], [7], [8]. Furthermore, women show more prefrontal involvement during cognitive control tasks [9], [10], however, not consistently [11]. Similarly, such sex differences along the anterior-posterior axis has been shown for visuospatial tasks, where women show higher prefrontal involvement [12]–[15], while men sometimes exhibit a stronger activation in parietal [14], [15] or primary sensory [12] regions. Sex differences in performance of visuospatial abilities can also partly be accounted for by differences in working memory capacity [16].

Cognitively demanding tasks typically activate a fronto-parietal network as assessed with functional magnetic resonance imaging (fMRI) [17]. Frontal regions are particularly associated with top down control and goal directed behavior [18], and parietal regions with sensory integration and attention [19]. Prefrontal and lateral parietal areas are connected through the longitudinal fasciculus, and the activity in this network has been associated with attentional control and working memory load [17]. The fronto-parietal cortical network can be observed bilaterally independent of stimulus modality [20], or lateralized to the left and right hemispheres depending on the respectively verbal and visuospatial nature of the task [21]–[24].

Task-related fMRI has been the traditional method of studying functional brain networks. However, recent years of research has established that the various sensory, motor, and cognitive networks can also be studied during rest with resting state fMRI (rs-fMRI). Activity of distributed cortical areas engaged in the same network oscillate in phase on a low frequency range (<0.1 Hz) also during rest. This allows identifying and separating individual networks through temporal correlation techniques [25], [26]. Applying this approach also allows to identify the fronto-parietal control network [27]–[29]. Frequently referred to as the task positive network, it has been suggested to be equivalent to the fronto-parietal network involved during tasks, and also has been shown to reorganize in response to a working memory task [30].

A number of recent studies have addressed sex differences in rs-fMRI. While one study did not detect any sex differences in default mode, salience, and (fronto-parietal) cognitive control network [31], other studies found sex differences in various sensory, motor, sub-cortical, or cognitive networks such as default mode, cognitive control, and language networks [26], [29], [32]–[36]. Filippi et al. [29] suggest, however, that sex differences are more evident within cognitive rather than sensory networks. Several of the above mentioned studies find women to have higher connectivity in prefrontal regions relative to men [32], [37], including for fronto-parietal networks [29]. However, Allen et al. [32] and Weissman-Fogel et al. [31] suggest no sex differences in fronto-parietal networks. A few studies also investigated sex differences in the laterality of resting state networks with conflicting results. Whereas Liu [34] found men to be more lateralized in both left and right lateralized systems, Filippi [29] report women to be more lateralized in default mode and attention networks. Applying graph theoretical approaches, Tomasi and Volkow [35], and Tian [36] found that women show higher functional connectivity in the left hemisphere, while men are more right lateralized. This also includes prefrontal regions. The consistency across findings is thereby low, and the diversity of methods and networks explored challenges the comparability across studies and groups of participants.

As implied by the terminology, rs-fMRI can dichotomously be referred to as the counterpart of the mind-state engaged during task execution (task-related fMRI). However, because of the unconstrained nature of resting state, there is also an on-going discussion of the degree of stability/variability in resting state. In other words, it is unclear to what extent rs-fMRI can be considered as a trait measure of a person rooted in underlying structural characteristics or more dependent on the current mind state of the person being tested. Evidence for the first view comes from studies that revealed a link between resting state functional connectivity and white matter pathways in the brain, including the fronto-parietal network [38], [39]. Further, test-retest reliability in resting state appears to be medium [40]–[42] to high [42]–[44], depending on methods and networks studied, which confirms a certain degree of stability across measurements, but also leaves 20–50% of variability unexplained. Arguments for state dependency comes from a range of studies suggesting rs-fMRI to be influenced by a number of variables such as task execution prior to rs-fMRI [45], time of day [46], or mood [47].

With respect to sex differences in resting state, studies seem to imply that they are a result of fixed and invariant sex differences in structural and functional connectivity, and therefore being a trait characteristic of the male and female brain. However, other studies have shown that the functional connectivity during task performance can change dynamically, for example with the hormonal state of female participants (e.g. menstrual cycle phase). Specifically, it has been shown that sex hormones change dynamically the functional cerebral organization by modulating hemispheric asymmetries and interhemispheric interaction across the menstrual cycle. Women tested behaviourally in the follicular and/or luteal phase, with respectively high levels of estradiol and/or estradiol and progesterone, are less lateralized as compared to the menstrual phase [48]–[51], and also show reduced functional connectivity between hemispheres [52]–[56]. In addition, estradiol appears to modulate cognitive control as assessed by cognitive inhibition [57], [58] and working memory tasks [59]–[61]. One of these studies [60] also showed that this estradiol-related modulation occurred in prefrontal and parietal regions. It should be highlighted that participants of the current cohort (data collected during the same sessions) showed an estradiol-related increase in cognitive control in the follicular phase as compared to menstrual and luteal phase [62]. Whether this estradiol effect is only task-related, or relies on changes in the intrinsic functional connectivity during resting state, is yet to be investigated.

The present study focuses on trait versus state aspects of sex and sex-hormonal differences in intrinsic functional connectivity in fronto-parietal networks. The fronto-parietal networks are particularly interesting due to potential sex differences in functional brain organization related to these networks, and menstrual cycle effects found previously in a cognitive control task for the current cohort (see above). Thus, the aim of the present study is twofold. First, we aim to investigate sex differences in rs-fMRI across three sessions, while controlling for sex-hormonal fluctuations across the menstrual cycle in women. According to previous resting state studies on cognitive control networks in general [29], [32], [37], and fronto-parietal networks in particular [29], it is expected that women will show higher prefrontal connectivity as compared to men. Based on task-related fMRI studies [5], [but see also 6], [63] showing men to be more lateralized than women, we also expect similar sex differences in rs-fMRI networks. Second, the current study aims to investigate whether resting state connectivity changes dynamically within short-term periods across the menstrual cycle. In line with the previously reported increase in cognitive control in follicular women of the same cohort [62], we predict estradiol-related changes in the prefrontal resting state connectivity in the follicular phase as compared to menstrual and luteal phase, whereas rs-fMRI is predicted to be more stable across corresponding time intervals in men.

## Materials and Methods

### Ethics statement

The study was approved by the Regional Committee for Medical Research Ethics (REK vest) at the University of Bergen. Participants gave their written informed consent according to the Declaration of Helsinki. Participants were financially compensated for their participation.

### Participants

Sixteen healthy women (out of twenty-one originally tested, see below for inclusion criteria) (mean ± SD: 23.25±5.01 years) and fifteen healthy men (23.13±2.42 years) completed three sessions of resting state fMRI. The sex of the participants was ascertained by self-report. All participants were native Norwegian speakers, and right handed (laterality quotient 93.33±11.16 for women and 93.78±10.23 for men) [64]. The women were tested once in three different cycle phases, i.e. the menstrual phase (day 2–4), the follicular phase (day 8–12), and the luteal phase (day 20–22). To estimate womens' cycle phases, individual length of the menstrual cycle was taken into account. Individual cycle length was calculated as an average of three consecutive cycles. Some women had used period calendars for several months before taking part in our study. The remaining women were followed for 3–4 months before the MRI scan. To estimate individual cycle phases, the back-counting method was applied. Self-reported onset of menses was used as a starting point. From this date, and by considering individual cycle length, the next menstruation-onset could be predicted. By counting back from this anticipated start of the next cycle, occurrence of the follicular and luteal phase could be predicted (e.g. for a 28-days cycle, this means counting back 17–21, and 7–9 days to capture follicular and luteal phase, respectively). Additional inclusion criteria for women involved a regular menstrual cycle with a mean cycle length of 26–32 days; no use of hormonal contraceptives or other hormone regulating medicaments currently or for the last six months; no pregnancy for the last six months prior to the study. To control for influence of circadian rhythm, time of testing deviated no more than three hours between testing sessions. To control for a possible session effect, women were randomized according to cycle phase at the first session (i.e. one third of the female sample started in each of the respective cycle phases. Men were tested three times with one to two weeks in between two testing sessions, and thereafter assigned into three groups equivalent to the female cycle phase groups. In addition to the resting state, a lexical decision task and a left-right confusion task was administered. The order of the three functional scans was randomized across subjects and sessions.

### Hormone assays

Two saliva samples were collected during each session for all participants, one before the resting state fMRI scan, and one after. An independent hormone laboratory completed the saliva analysis by applying luminescence assays on an average amount of the two samples. Analysis was done for concentration of estradiol and progesterone.

Sixteen women were included for subsequent statistical analysis. Luteal progesterone levels served as an indicator of ovulation in all women, which again served as basis for inclusion. Estradiol and progesterone levels were within the expected range for the respective cycle phases (see Table 1). A repeated measures ANOVA was done on progesterone levels and revealed a significant effect of cycle phase (F(2,30) = 37.8, p<0.001, η^2^ = 0.72). Fishers LSD post-hoc test showed a significant differences between the menstrual and luteal phase (p<0.001), and between the follicular and luteal phase (p<0.001). The same ANOVA on estradiol levels also revealed a cycle phase effect (F(2,30) = 6.48, p = 0.004, η^2^ = 0.3). Fishers LSD post-hoc analysis revealed a significant difference between the menstrual and luteal phase (p = 0.001), while the difference between the follicular and luteal phase marginally failed to reach statistical significance (p = 0.06). Of the remaining sixteen women, six started testing in their menstrual phase, five in their follicular phase, and five in their luteal phase. Estradiol and progesterone levels of men were not tested because these two gonadal steroid hormones are known to be very low, and close to the detection limit of the hormone assays.

**Table 1:** Means, standard deviations, and range (in brackets) in estradiol and progesterone levels from saliva samples in the women (n = 16) during the menstrual, follicular and luteal cycle phase.

Hormone in pg/ml	Menstrual	Follicular	Luteal
Estradiol	2.7±1.3 (1.3–5.3)	3.6±1.5 (1.6–6.3)	4.5±1.6 (2.1–7.7)
Progesterone	53.2±17.8 (25.2–1.5)	57.3±30.4 (23.6–136)	191.4±93.8 (95.2–416.7)

### Resting state fMRI

The participants completed three sessions of rs-fMRI. They were instructed to relax and keep their eyes closed during scanning. The data were collected with a 3T GE-Signa MRI scanner. An anatomical T1-weighted image was acquired prior to the fMRI for each subject at each session (3DT1 FSPGR, TR/TE/FA/FOV 7.9 ms/3.2 ms/11°/256 mm, 256×256 scan matrix, 180 sagittal slices, voxel size 1×1×1 mm). For the functional images a gradient-echo echo-planar imaging (GE-EPI) sequence was used. 150 images were collected for each session with whole brain coverage (TR/TE/FA/FOV 2800 ms/30 ms/90°/128 mm, 128×128 matrix, 35 axial slices, voxel size 1.72×1.72×3.5 mm).

### Data analysis

The first three scans were treated as dummy scans and were rejected in the subsequent analysis. Prior to the statistical analysis the data went through pre-processing in SPM8 software (Welcome Trust Centre for Neuroimaging, www.fil.ion.ucl.ac.uk) implemented in Matlab R2009a (The MathWorks, Inc., Natick, MA, USA, www.mathworks.com). Preprocessing involved the steps realignment (reference volume: the first EPI volume obtained), and unwarping, normalization of the anatomy (template image provided by the Montreal Neurological Institute (MNI)), resampling with a voxel size of 4×4×4 mm, and smoothing (FWHM 8 mm).

GIFT [Group ICA of fMRI Toolbox; Version 1.3i; 65] was used for a group level Independent Component Analysis (ICA). In a first preprocessing step, the individual data was mean corrected, by subtracting the image mean per time-point. Thereafter, following the GIFT default settings, three analysis steps were applied. First, the data went through a data reduction step using principal component analysis (PCA). This was done separately for each participant to reduce individual data dimensionality. Afterwards the individual data were group-concatenated and then subjected to another two PCA data reduction steps. Second, the reduced data were used for estimation of forty independent components using the infomax algorithm. The third step involved back-reconstruction, using GICA, of individual spatial maps from the components estimated at group level. The values of each participant's maps and time courses were scaled to represent percent signal change. No temporal filtering was applied on the data in GIFT.

**Figure 1:**
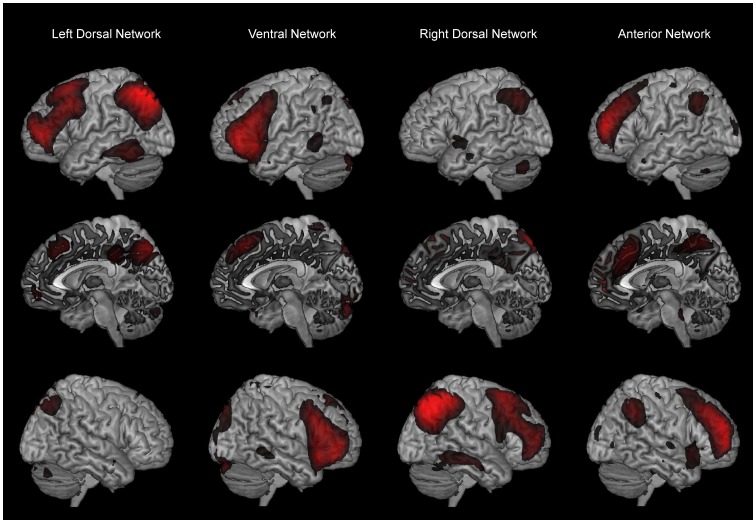
Spatial maps of independent components. Depicted in left, medial and right view are the four independent components selected for further statistical analysis: left dorsal; ventral; right dorsal; anterior network.

Spatial sorting was used to identify fronto-parietal networks among the forty components. The degree in which these networks are lateralized varies in the resting state literature [27], [66]. However, we chose to construct the sorting templates in line with Corbetta et al. [67] who distinguishes between left and right networks, and networks of more dorsal and ventral localization. Thereby the components were consecutively spatially sorted after four templates, and only the component with the highest concordance with the respective templates was selected for the subsequent group statistic. The four templates comprised the following regions: for dorsal networks (left or right) inferior parietal lobe (IPL), superior parietal lobe (SPL), middle frontal gyrus (MFG), and precentral gyrus (PCG); for ventral networks (left or right) supramarginal gyrus (SMG), superior temporal gyrus (STG), MFG, inferior frontal gyrus (IFG) triangularis and operculum [67]. This procedure resulted in four spatially distinct networks (see Figure 1). The dorsal networks were clearly lateralized and correlated with left (r = 0.40), and right dorsal templates (r = 0.33). The two ventral networks were bilaterally organized. One of these two ventral networks correlated strongest with left ventral template (r = 0.31), while the other one was identified as the anterior fronto-parietal network and correlated strongest with right ventral template (r = 0.23). Corresponding individual spatial maps for each component were then further explored in terms of sex and menstrual cycle phase effects, using SPM8 second level statistics. In the following, these four networks will be referred to as the left dorsal, right dorsal, ventral, and anterior network.

Group analyses of the spatial maps for each of the four components were estimated using the individual back-reconstructed ICs. This allowed to investigate whether the connectivity amplitude between the networks of the components and overall brain regions (including hypothesized regions) vary according to sex or cycle phase. As the whole brain is represented in the spatial maps, the analysis estimates statistical parameter maps both for voxels within the core region of the network (component) as well as for voxels in distant, no-core regions. For each component two types of analysis were carried out. First, a 2 (Sex) ×3 (Cycle Phase) ANOVA using the full factorial design setup, implementing the factors Sex (levels specified as being independent and unequal in variance) and Cycle Phase (levels specified as dependent with equal variance). Total brain volume was implemented as a covariate to control for differences in brain size between men and women. Estimates of individual brain volume were calculated from tissue probability maps in subject space, generated from each individual's structural T1 image using unified segmentation and normalization routines in SPM8. These maps were multiplied by the volume per voxel, summed across the entire imaged volume and between relevant tissue classes to obtain a final estimate of brain volume. To assure that movement does not affect the results, the individual realignment parameters were characterized by calculating four estimates of movement according to Van Dijk et al. [68]. The estimates were mean translation, maximum translation, total translation, and mean rotation [see 68 for calculation procedure]. Mean translation and mean rotation were also included as regressors in the ANOVAs. The results were explored at a significance threshold of p<0.00025, adjusted for multiple testing, and a cluster threshold of minimum 10 contiguous voxels. The F-contrasts from these ANOVAs were also subjected to effect size analyses using the ImCalc function in SPM8. Effect size measures were expressed as ω^2^ = (f_2_/(1+f_2_)), with f_2_ = ((df_num_*(F_emp_−1))/n_obs_) (in which the abbreviations refer to degrees of freedom numerator; empirical; observations). Results from these analyses are found in (Figure S1 and S2). Second, to explore the data further, a multiple regression for each of the cycle phases including the regressors estradiol, progesterone, and the interaction of estradiol and progesterone. The multiple regression results were explored at a significance threshold of p<0.001 and a cluster threshold of minimum 10 contiguous voxels. The less conservative statistical threshold was chosen to increase statistical power, in particular with regard to potential sex hormonal effects across cycle phases. The corresponding statistical maps were explored with MRIcron (www.mricro.com, version 6/2013

To specifically quantify test-retest reliability, we calculated an intraclass correlation (ICC) analysis on the individually back-reconstructed spatial maps from the group ICA for each component separately for men and women [69]. The ICC analysis was done voxel-wise, and estimates the difference of within-subject variability across the three sessions per subject (MSW), and between-subject variability (MSB). Thus, the ICC represents the proportion of total variance within the data that is explained by the variance between the testing sessions: ICC = (MSB−MSW)/(MSB+2MSW). ICC ranges from 0–1, and the closer one approaches 1 the more is the observed variance explained by the between-subject variance rather than within-subject variance, indicating a higher test-retest reliability for the given sample of subjects.

To estimate the size of menstrual cycle effects detectable within the current sample size, an a posteriori power analysis was conducted (G*Power 3.1.3.: http://www.psycho.uni-duesseldorf.de/abteilungen/aap/gpower3). Also, regional differences in gray matter were explored a posteriori, using voxel-based morphometry (VBM). Therefore, the T1 weighted images were segmented into gray- and white-matter maps and corrected for the effect of the spatial normalization (modulated maps). The normalized unmodulated maps were implemented in a 2 (Sex) ×3 (Cycle Phase) ANOVA as the aim was to explore gray matter regional sex-differences in particular. Corresponding significance threshold as for the multiple regressions (see above) was applied.

## Results

**Figure 2:**
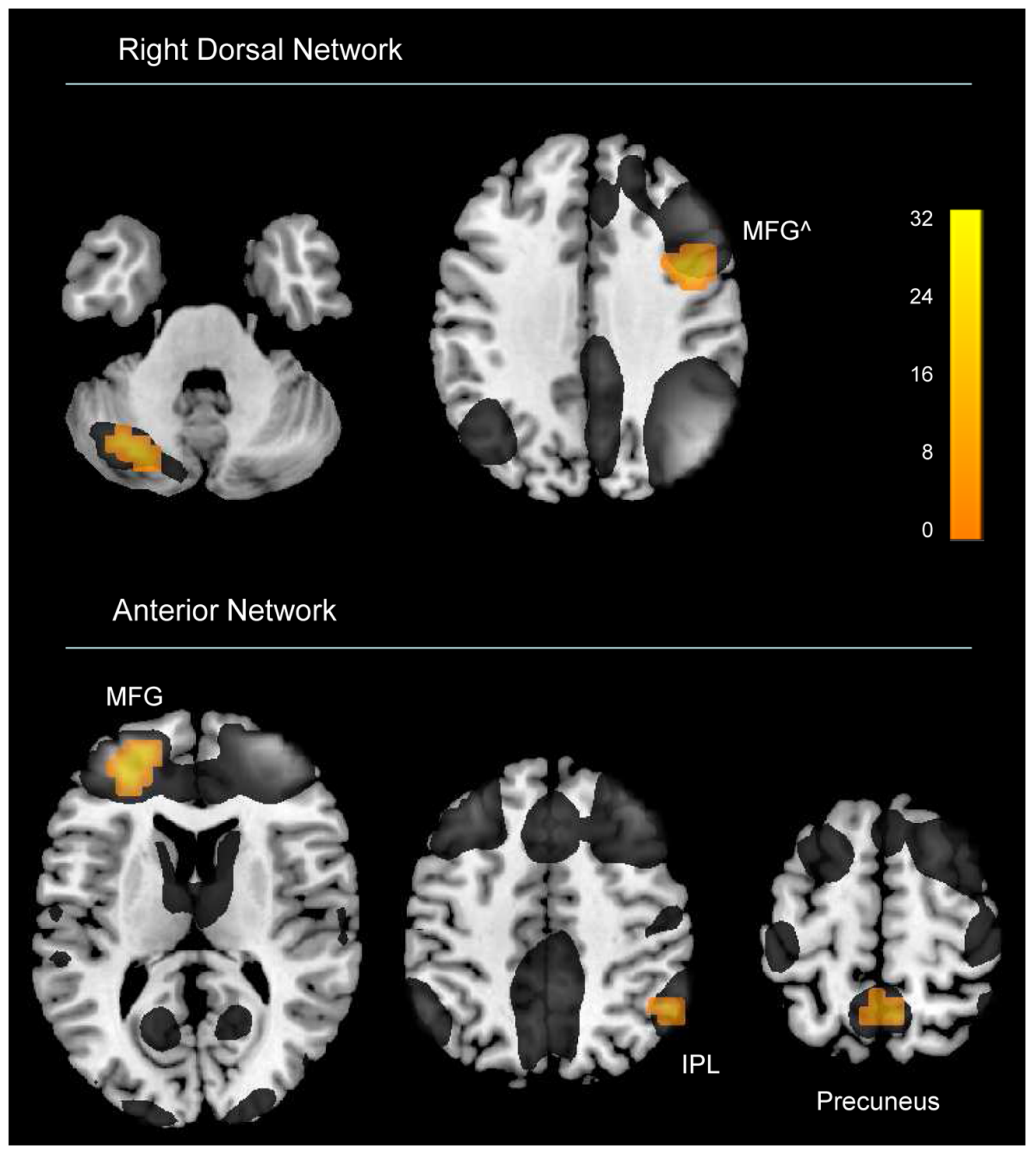
Sex differences in fronto-parietal networks. Main effect of sex was found for two networks. Results are uncorrected (p<0.00025), cluster size 10, projected onto a standard brain template. Right dorsal network (z = −35, 36); Anterior network (z = 12, 48 and 62). Yellow blobs represent areas of higher connectivity for women relative to men; gray represents overlaid maps of the respective independent components. Abbreviations: MFG – middle frontal gyrus, IPL – inferior parietal lobe. ∧ significant when brain volume correction is left out.

**Figure 3:**
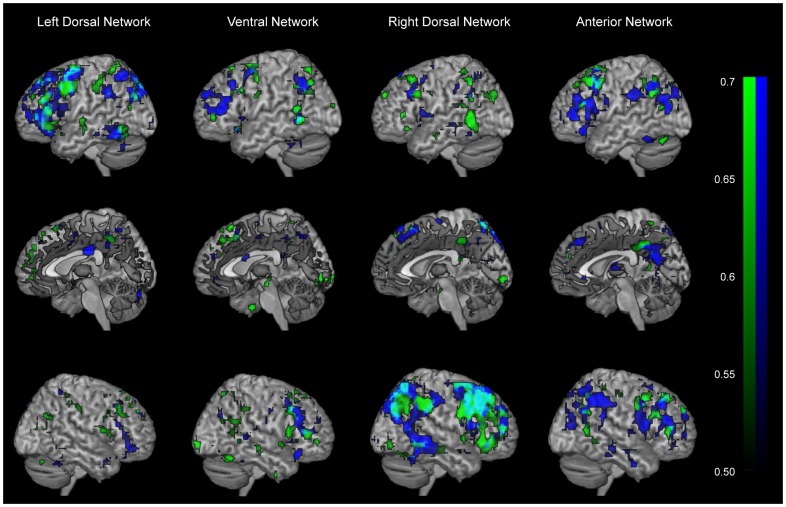
Test-retest reliability analysis. Rendered ICC maps in left, medial, and right hemispheric view for men (blue) and women (green) calculated for the four frontoparietal networks: Left dorsal; Ventral; Right dorsal; Anterior network. The overlapping ICC maps between men and women are shown in turquoise. Depicted are voxels which survived the correlation threshold of r = .50. The colour range represents correlational values from .50 (darkest) to .70 and above (lightest).

The 2 (Sex) ×3 (Cycle Phase) ANOVAs revealed main effects of Sex in the right dorsal, and the anterior network. No sex difference was found in the left dorsal or ventral network. For the right dorsal network women showed higher connectivity in left cerebellum. For the anterior network, women showed higher functional connectivity in left MFG, bilateral precuneus, as well as right IPL (see Figure 2, Table 2). No main effect of Cycle Phase/repeated measures, or interaction effect between Sex and Cycle Phase, was found in any of the networks. A sensitivity (power) analysis revealed given a power of 0.80, and p-level of 0.05, that medium to large effects can be excluded (η^2^ = 0.14) [70]. Neither of the multiple regression analyses for women only, using individual hormone levels as regressors, was significant. The total brain volume differed significantly between men and women (F(1,29) = 22.7; p<0.001; η^2^ = 0.44), though no effect of Cycle Phase/repeated measures or interaction between Sex and Cycle Phase was found. When total brain volume was not included as covariate in the analysis of the four components' spatial maps, the following changes with respect to sex differences were evident: for the right dorsal network, an additional region in the right MFG (31 voxels) was found showing higher connectivity in women. For the anterior network, the right IPL no longer showed higher connectivity for women. No sex differences were found for any of the estimated movement parameters. Also, excluding of the movement parameters as covariates from the ANOVAs, did not change the results significantly. The ICC analysis revealed medium to high reliability for men and women in overlapping areas (see Figure 3), which also follow the fronto-parietal localization of the components. The voxel-based morphometric analyses on modulated gray matter maps revealed no significant effects.

**Table 2:** Sex differences in resting state functional connectivity of fronto-parietal networks.

Network	Cluster size	F	Z		Coordinates		Side	Area	BA	Connectivity
				X	Y	Z				
Right dorsal network										
	17	25.18	4.53	−26	−72	−34	L	Cerebellum		Women>Men
	31	26.31* ^∧^	4.63	42	8	38	R	MFG	6	Women>Men
Anterior network										
	39	32.96*	5.13	−30	44	14	L	MFG	46	Women>Men
	10	23.74	4.41	50	−52	50	R	IPL	40	Women>Men
	13	19.19	3.98	6	−56	62	R	Precuneus	5	Women>Men

Note: Results for the two components that showed significant main effect of sex, i.e. right dorsal network, and anterior network. Abbreviations: BA – Broadmann area, L – left, R - right, MFG – middle frontal gyrus, IPL – inferior parietal lobe.

*peak voxel survives FWE correction.

∧ significant only when brain volume correction is not implemented.

The raw-data used in analysis are publicly available at http://fmri.uib.no/data/rsfmri-gender. The F-maps, ICC maps, and effect size maps are available at http://neurovault.org/collections/56/.

## Discussion

The study investigated sex differences and menstrual cycle effects in four fronto-parietal networks (see Figure 1) in a repeated measures design. Two of these networks showed sex differences, comprising the right dorsal network and the anterior network (see Figure 2, Table 2). For these two networks, women showed generally higher functional connectivity, and particularly in prefrontal regions. No menstrual cycle effects were found. Reliability maps show medium to high reliability for both men and women (see Figure 3).

### Trait and state aspects of sex (hormonal) differences in resting state

As to what degree resting state activity is stable across testing sessions or susceptible to mind states of the participant is debated in the literature. The current study further investigated stability versus variability by using sex as trait measure and hormonal fluctuations across the menstrual cycle as state measure. The main effect of sex (together with the non-significant Sex by Cycle Phase interaction) in fronto-parietal networks (see Figure 2, Table 2) argues for stable sex differences in resting state across time. These findings also suggest that sex differences in resting state occur independently of females' sex-hormonal state during different cycle phases. Moreover, it has been shown (see Figure 3) that the test-retest reliability of the ICC maps for all four components is medium to high in *both* men and women.

Sex-hormonal effects on the brain are broadly divided into two categories of organizing (trait) and activating (state) effects [71]–[73]. Although this distinction is not as clear-cut, organizing effects of sex hormones occur mainly early in ontogenesis [e.g. 71] or during puberty [74], and are believed to establish permanent sex differences in brain structure and corresponding functions. Activating effects of sex hormones are, however, transitory and mainly related to dynamic functional changes in the brain. Due to the fact that the present study found functional connectivity in fronto-parietal resting networks to be relatively stable across three sessions (and menstrual cycle phases) in both sexes, the results might suggest sexual dimorphisms in underlying white matter structure, which were not affected by activation effects of sex hormones. This also suggests that menstrual cycle effects reported in previous fMRI studies occur as a result of sex-hormonal modulation of task-related brain activity. It is less likely that sex hormones modulate the underlying functional connectivity that is task independent, at least for resting state networks that were investigated in the present study.

In fact, several studies suggest sex differences in white matter, also when brain size is controlled for [see 75, for a review]. Although women have generally shown smaller total white matter volume than men [76], a recent diffusion MRI tractography study revealed greater overall cortical connectivity in women after correcting for brain size [77]. For frontal regions in particular, women have shown higher functional anisotropy (FA) than men [78], also involving anterior parts of the corpus callosum [79], [ but also see 80]. In contrast, men revealed higher FA in the *left* superior longitudinal fasciculus relative to women [81]. Interestingly, an FA increase in the *right* superior longitudinal fasciculus was observed in female-to-male transsexuals after hormonal treatment [82]. However, how exactly these sexual dimorphisms in white matter relate to functional connectivity in resting state fMRI must be further explored in future studies. The current study argues though, that regional *gray matter* sexual dimorphisms are unlikely to underlie the sex differences found in resting state, as the gray matter maps analysis showed no sex-differences.

### Sex differences in fronto-parietal resting state networks

Women showed generally higher connectivity as compared to men, and the largest cluster was found in the left MFG for the anterior network. Another cluster is found in the right MFG for the right dorsal network when brain volume correction is left out. Several previous rs-fMRI studies report women to have higher connectivity in prefrontal regions for cognitive networks, including IFG [29], [32], MFG [29], [37] and medial prefrontal regions [29], [32], [37]. However, of all the studies that found sex differences in resting state, only three addressed fronto-parietal networks. Whereas Allen et al. [32], and Weissman-Fogel et al. [31] did not find sex differences, Filippi et al. [29] report higher connectivity for women in the right IFG, and left cerebellum for a network similar to the right dorsal network in the current study. They also reported higher connectivity in the right insula and cerebellar regions for a network similar to the left dorsal network of the current study. Further, Filippi et al. [29] found stronger connectivity for men in posterior regions (i.e., right STG and left IPL for the right dorsal network, and left SPL for the left dorsal network). In contrast to these findings, the current study did not detect regions of higher connectivity in men relative to women. However, in support of Filippi et al. [29] we found that women relative to men showed the strongest connectivity in prefrontal and cerebellar regions, though in addition parietal areas were found. As for the sex differences in MFG connectivity, it is also interesting to note that Zuo et al. [37] reported higher homotopic connectivity in this region for women.

Sex differences in functional brain organization of the prefrontal cortex have already been proposed in task-related studies, in which men and women also showed a behavioral difference. Neuroimaging studies have shown that in the visuospatial domain, women engage more prefrontal regions [12]–[15], while men activate more parietal [14], [15] or sensory [12] regions. Butler et al. [12] suggest that these sex differences in activation might indicate that men and women apply different strategies to identical cognitive problems. That is, women perform mental rotation tasks by more effortful “top-down” control, whereas men rely more on automatic “bottom-up” processes. Others have suggested, based on observed higher left prefrontal activation that women rely more on verbal strategies in mental rotation [83]. These sex differences in functional brain organization are only partly reflected in the current rs-fMRI study. Although we found higher functional connectivity in anterior and posterior regions in women, both task-related and rs-fMRI suggest higher functional activity and connectivity, respectively, in particular for prefrontal regions. For men, however, the current study did not find a clear link between task-related and rs-fMRI in posterior regions [but see 29]. Given that sex differences in resting state- and task-related fMRI involve similar brain regions and networks, it is possible that sex differences in resting state underlie some of the reported sex differences in task-related brain activation and behavior.

Another principal of functional brain organization is lateralization of cortical functions. Sex differences in lateralization in resting state are inconsistent. It is widely assumed that men are generally more lateralized than women in various cognitive abilities [3]–[5], [but see also 6]. In line with this observation, Liu et al. [34] suggest men to be more lateralized in both left and right lateralized systems. In contrast, Filippi et al. [29] suggested women to be more lateralized than men in default mode, and attention networks, but for fronto-parietal networks this study did not find any sex differences in lateralization. In the current study, women show higher connectivity in both left and right MFG, which might suggest commonalities with studies showing more bilateral activation and connectivity in the female brain. However given that these two findings belong to two different networks, this is rather speculative.

In addition to the stronger prefrontal functional connectivity in women, the anterior fronto-parietal network shows higher connectivity in the precuneus. This parietal region is suggested to be a core region in the default mode network [84]. The activity in the default mode network exhibits an anti-correlation with the activity in the task-positive fronto-parietal network [85], [86]. The current results might thereby imply less anti-correlation between the fronto-parietal network and the default mode network in women as compared to men. Similarly, Bluhm et al. [33] have found higher connectivity for women relative to men between the default mode network, and prefrontal and parietal regions (ICA). This study also found higher connectivity within default mode regions in women (seed-based analysis). The former finding might suggest closer communication between fronto-parietal and default mode areas in women. In contrast to the task-positive network, the default mode network is associated with inwards direction of attention, such as engagement in self-referential thoughts [87] and episodic memory [88].

### Lack of menstrual cycle effect

This study investigated the activating effects of sex hormones on rs-fMRI, and whether the partly conflicting findings regarding sex differences in rs-fMRI depend on female participants' sex-hormonal state. We have previously shown estradiol-related changes in cognitive control across the menstrual cycle in the same cohort [62], and we therefore focused particularly on the intrinsic functional connectivity in fronto-parietal resting state networks. However, no menstrual cycle effect or hormone-rs-fMRI correlations were found.

The lack of menstrual cycle effects suggests that the previously observed hormonal effects in cognitive control may depend more on actual task execution rather than on intrinsic functional connectivity during resting state. In fact, previous fMRI studies found task-related changes in frontal and parietal brain activity in relation to sex hormones [60], and task-related connectivity changes in frontal and parietal brain activity in relation to sex hormones [56], [89]. Also during rest, previous studies report menstrual cycle related changes in PFC in glucose metabolism [90], alpha asymmetry [91], and glutamate levels [92]. These results might indicate that resting state network connectivity, in particular, is unaffected by sex hormones. However, the lacking menstrual cycle effect in the current study might also be partly due to a methodological issues. Given that rs-fMRI relies on the detection of low frequency BOLD signal fluctuations, it may be less sensitive to state changes as compared to task-fMRI. Damoiseaux et al. [93] have shown that for healthy subjects, rs-fMRI is consistent across subjects and sessions for a number of networks, including the executive functioning. It is therefore reasonable to assume that rs-fMRI in its current application is not sensitive enough to reliably detect hormone level related changes in functional connectivity. Furthermore, we cannot rule out that other than fronto-parietal resting state networks are sensitive to sex-hormonal changes, or that it is rather the inter-network connectivity that are affected, and/or that other sex hormones (e.g., testosterone) exert transient influences on resting state connectivity.

It is important to note, however, that a recent rs-fMRI study [94] found menstrual cycle-related effects on intrinsic functional connectivity in default mode, and also in the executive control network comparable to fronto-parietal networks investigated in the current study. Specifically, Petersen et al. [94] found higher connectivity in the right anterior cingulate region for women tested in the menstrual phase (which they refer to as ‘early follicular’) as compared to the luteal phase, as well as higher connectivity in the left MFG in the menstrual phase as compared to women taking hormonal contraceptives. Despite of several methodological similarities between this and the current study, there were also some differences that might partly account for the conflicting findings. For instance, the preprocessing procedures differed leading to presumably stricter thresholding of our results. In addition, the power might be higher in Petersen et al.'s study as it includes data from a larger sample (20 menstrual phase, and 25 luteal phase). However, women in the menstrual phase (cycle day 2 to 6) in Petersen et al.'s study revealed physiologically unusually high progesterone levels (ca. 100 pg/ml, as compared to 53.2 pg/ml (cycle day 2 to 4) in the current study). This also resulted in relatively small differences in progesterone level between the menstrual and luteal phase of about 40 pg/ml, as compared to 138 pg/ml in the current study. Given that Petersen et al. did also not find significant cycle-related difference in estradiol levels, it is puzzling whether all women in Petersen et al.'s study were tested in the correct cycle phase. In other words, the current study should have been even more likely to find sex hormonal effects on rs-fMRI, if this effect really exists. However, this was obviously not the case. Therefore, it should be considered that the effect of Petersen may be due to some cycle-unrelated differences between the groups (e.g. differences in personality traits) as a between-subjects design was used. Such factors are better controlled by repeated measures design. Finally, it should be noted that Petersen et al. tested a sample of women only (between-subject design) while the current study tested a sample of female and male participants three times in a repeated measures design. Although it is unclear whether the inclusion of male control group can account for the conflicting findings between studies, it has been shown to provide important baseline information of random variability in repeated measure rs-fMRI. As visualized in the effect size maps (see Figure S1 & S2), the effect sizes for cycle-related fluctuations in rs-fMRI were similarly small as they were for rs-fMRI fluctuations between the male groups.

## Conclusions

The current study investigated sex differences and menstrual cycle effects in resting state functional connectivity of fronto-parietal cognitive control networks. Women showed generally higher functional connectivity, including in prefrontal regions, as compared to men. However, no menstrual cycle effects were found. The implications of these findings are multiple. First, the sex differences found in functional brain organization in fronto-parietal networks show similarities to those reported for task-related fMRI (e.g., visuospatial tasks), and might underlie at least partly sex differences in brain activation and behavior. Second, the lack of menstrual cycle effects suggest that sex hormones can be linked to task execution rather than hormonal modulation of underlying resting state connectivity. However, this needs to be investigated in future rs-fMRI studies, and requires to directly compare sex and sex-hormonal effects of task-fMRI and rs-fMRI. Finally, in spite of the unconstrained nature of rs-fMRI (i.e. not restricted by task), the current study revealed stable resting state networks in *both* men and women, indicating that rs-fMRI is generally a reliable technique, and further suggests that *resting states* can be considered as *resting traits*.

## Supporting Information

Figure S1 **Effect size maps.** Depicted are effect size maps from the ANOVAs conducted on the spatial maps of the components. Main effect of Sex and Cycle Phase/Repeated measures (note that this is across sex, so the randomized male groups are also included), and interaction of Sex and Cycle Phase are shown for the Left dorsal network, and the Ventral network. To show the effect sizes in women cycle phase groups separately from the groups in men, results from one-way ANOVAs are included. Effect sizes are calculated as ω^2^, and depicted in a colour range from blue (low effect size) to green (higher effect size). Effect size maps are available at http://neurovault.org/collections/56/.
(TIF)

Figure S2 **Effect size maps.** Depicted are effect size maps from the ANOVAs conducted on the spatial maps of the components. Main effect of Sex and Cycle Phase/Repeated measures (note that this is across sex, so the randomized male groups are also included), and interaction of Sex and Cycle Phase are shown for the Right dorsal network, and the Anterior network. To show the effect sizes in women cycle phase groups separately from the groups in men, results from one-way ANOVAs are included. Effect sizes are calculated as ω^2^, and depicted in a colour range from blue (low effect size) to green (higher effect size). Effect size maps are available at http://neurovault.org/collections/56/.
(TIF)
