# Supplementary figures and images for: Resting States Are Resting Traits – An fMRI Study of Sex Differences and Menstrual Cycle Effects in Resting State Cognitive Control Networks
Source: PLoS One. 2014 Jul 24;9(7):e103492. doi: 10.1371/journal.pone.0103492 (PMC4110030; doi:10.1371/journal.pone.0103492)

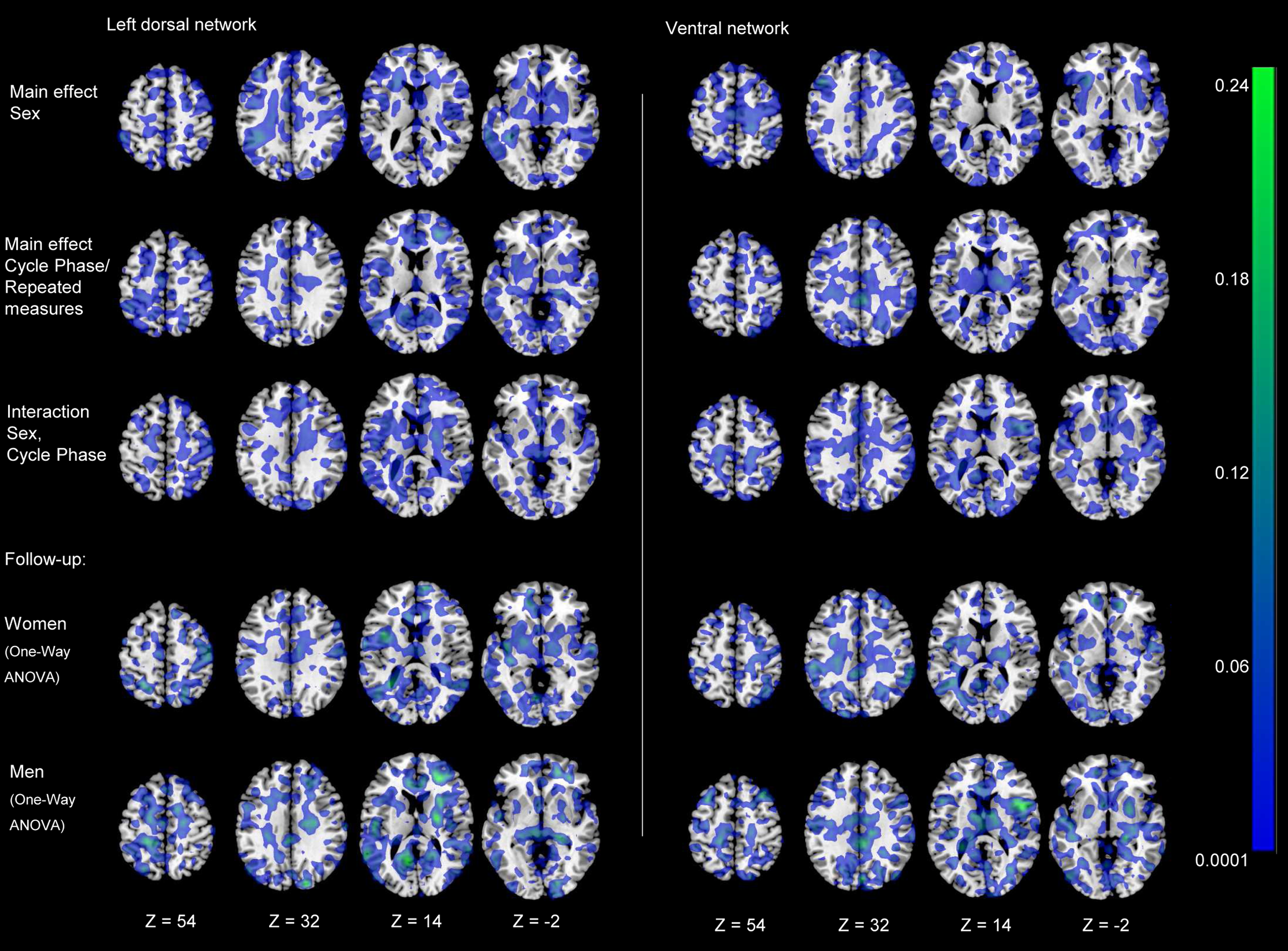

Supplement: Figure S1 — Effect size maps. Depicted are effect size maps from the ANOVAs conducted on the spatial maps of the components. Main effect of Sex and Cycle Phase/Repeated measures (note that this is across sex, so the randomized male groups are also included), and interaction of Sex and Cycle Phase are shown for the Left dorsal network, and the Ventral network. To show the effect sizes in women cycle phase groups separately from the groups in men, results from one-way ANOVAs are included. Effect sizes are calculated as ω2, and depicted in a colour range from blue (low effect size) to green (higher effect size). Effect size maps are available at http://neurovault.org/collections/56/. (TIF) [file pone.0103492.s001.tif]

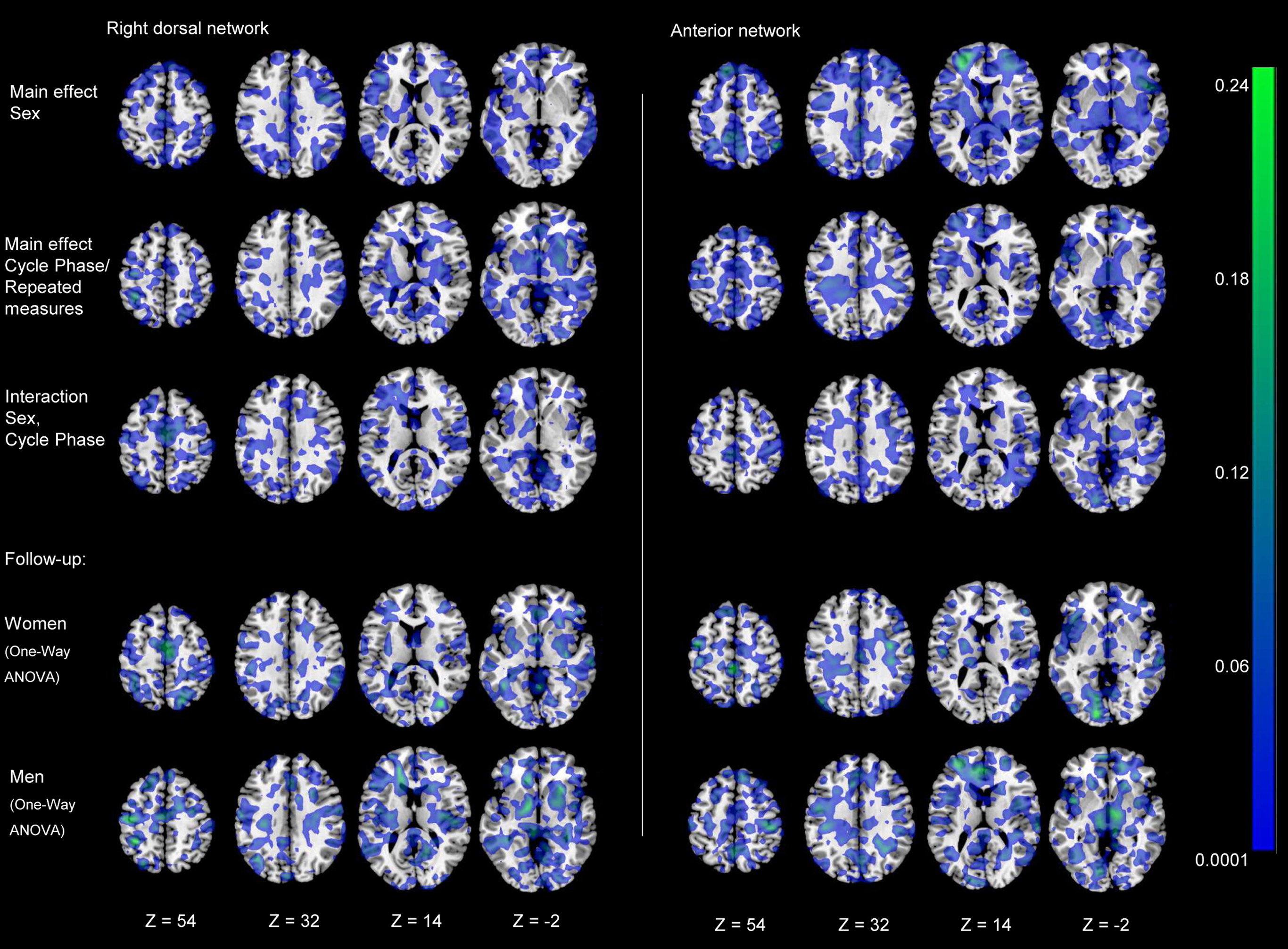

Supplement: Figure S2 — Effect size maps. Depicted are effect size maps from the ANOVAs conducted on the spatial maps of the components. Main effect of Sex and Cycle Phase/Repeated measures (note that this is across sex, so the randomized male groups are also included), and interaction of Sex and Cycle Phase are shown for the Right dorsal network, and the Anterior network. To show the effect sizes in women cycle phase groups separately from the groups in men, results from one-way ANOVAs are included. Effect sizes are calculated as ω2, and depicted in a colour range from blue (low effect size) to green (higher effect size). Effect size maps are available at http://neurovault.org/collections/56/. (TIF) [file pone.0103492.s002.tif]
